# Utility of quantitative MRI metrics in brain ageing research

**DOI:** 10.3389/fnagi.2023.1099499

**Published:** 2023-03-09

**Authors:** Pavel Filip, Viktória Kokošová, Zdeněk Valenta, Marek Baláž, Silvia Mangia, Shalom Michaeli, Lubomír Vojtíšek

**Affiliations:** ^1^Department of Neurology, First Faculty of Medicine, Charles University and General University Hospital, Prague, Czechia; ^2^Center for Magnetic Resonance Research (CMRR), University of Minnesota, Minneapolis, MN, United States; ^3^Department of Neurology, Faculty of Medicine, Masaryk University and University Hospital Brno, Brno, Czechia; ^4^First Department of Neurology, Faculty of Medicine, University Hospital of St. Anne, Masaryk University, Brno, Czechia; ^5^Department of Statistical Modelling, Institute of Computer Science of the Czech Academy of Sciences, Prague, Czechia; ^6^Neuroscience Centre, Central European Institute of Technology (CEITEC), Masaryk University, Brno, Czechia

**Keywords:** ageing, quantitative MRI, rotating frame relaxometry, diffusion weighted imaging, resting state functional MRI

## Abstract

The advent of new, advanced quantitative MRI metrics allows for *in vivo* evaluation of multiple biological processes highly relevant for ageing. The presented study combines several MRI parameters hypothesised to detect distinct biological characteristics as myelin density, cellularity, cellular membrane integrity and iron concentration. 116 healthy volunteers, continuously distributed over the whole adult age span, underwent a multi-modal MRI protocol acquisition. Scatterplots of individual MRI metrics revealed that certain MRI protocols offer much higher sensitivity to early adulthood changes while plateauing in higher age (e.g., global functional connectivity in cerebral cortex or orientation dispersion index in white matter), while other MRI metrics provided reverse ability—stable levels in young adulthood with sharp changes with rising age (e.g., T1ρ and T2ρ). Nonetheless, despite the previously published validations of specificity towards microstructural biology based on cytoarchitectonic maps in healthy population or alterations in certain pathologies, several metrics previously hypothesised to be selective to common measures failed to show similar scatterplot distributions, pointing to further confounding factors directly related to age. Furthermore, other metrics, previously shown to detect different biological characteristics, exhibited substantial intercorrelations, be it due to the nature of the MRI protocol itself or co-dependence of relevant biological microstructural processes. All in all, the presented study provides a unique basis for the design and choice of relevant MRI parameters depending on the age group of interest. Furthermore, it calls for caution in simplistic biological inferences in ageing based on one simple MRI metric, even though previously validated under other conditions. Complex multi-modal approaches combining several metrics to extract the shared subcomponent will be necessary to achieve the desired goal of histological MRI.

## Introduction

Over the course of ageing, human brains undergo extensive remodelling, a combination of accumulating damage and reparatory processes affecting structure, microstructure and functional patterns. Be it cellular loss, degenerative changes of myelin sheaths, alterations of iron metabolism, accumulation of neurofibrillary tangles or shifts in biochemical processes (Yankner et al., 2008), it’s exceedingly difficult to get a complex, but still non-invasive insight into these processes.

The versatility of MRI has emerged as a viable candidate—the combination of MRI protocols with different sensitivities allows us to exploit the relative contributions of various histological features of tissue. Nonetheless, the true linkage of MRI signal to the underlying biological system poses an exceptional challenge due to its elaborate, complex nature. The multitude of various cell types, their densely packed axons and dendrites of immense importance for the cerebral function despite their small diameters, astrocytes and terminal vasculature are well beyond the current resolution limits of *in vivo* MRI in humans. The MRI signal from an imaging voxel is therefore an inherently aggregated measure of the constituent parts, where different MRI protocols provide a different sensitivity to primary contrast drivers as macromolecules—mainly myelin (Stüber et al., 2014), cell membranes (Palombo et al., 2020) and paramagnetic iron (Bilgic et al., 2012; Möller et al., 2019). All of these are expected to be closely linked with ageing.

Furthermore, true assessment of normal biological processes, pathologies or even responses to therapeutical interventions intrinsically requires the extraction of quantifiable measures—quantitative imaging biomarkers (Kessler et al., 2015), going beyond the conventional MRI aimed at local anatomical contrast. Ideally, these quantitative MRI (qMRI) parameters should provide calibrated measures directly comparable across time points and imaging sites (Zaidi, 2006). A large number of MRI modalities hypothesised to meet these criteria are available. The measurement of water proton relaxation time/rate constants is one of the most straightforward approaches, providing quantitative information on molecular constituents in the micro-environment and also water molecule movement between different micro-environments (Does, 2018). Diffusion weighted imaging (DWI) offers a wide range of applications from reconstructing white matter pathways to estimating cell sizes and evaluating the axonal integrity (Dyrby et al., 2018). Myelination has been the focus of multiple targeted protocols. The rather controversial, but easy-to-acquire ratio of T1-weighted (T1w) and T2-weighted (T2w) signal has been labelled as “myelin map” based on the strong spatial correlation with histologically-derived myelin maps (Glasser and Van Essen, 2011). More advanced measures as magnetization transfer (Schmierer et al., 2004) and non-adiabatic RAFF4 (Relaxation Along a Fictitious Field of rank 4; Liimatainen et al., 2010) have been validated directly against histological samples. And last, but not least, parameters as volumes of specific structures of interest and certain “semiquantitative” measures of resting-state functional MRI (rs-fMRI) with an extensive body of research backing their utility may be also considered of value in this context.

Ergo, the benefits of multi-modal MRI acquisitions are more than apparent despite the time requirements and costs since they provide multifaceted and complementing views of the under-pinning biological structures (Castiglioni et al., 2019; Zhang et al., 2020; Weiskopf et al., 2021). The presented cross-sectional study was designed to capitalise exactly on this quantitative multiparameter mapping approach, consisting of:

– three relaxometry metrics:
  o adiabatic longitudinal relaxation constant T1ρ as a measure of cellular density (Michaeli et al., 2009)
  o adiabatic transverse relaxation constant T2ρ as a measure of iron level (Mitsumori et al., 2009)
  o non-adiabatic RAFF4 as a measure of myelin (Liimatainen et al., 2010)
– two DWI metrics based on NODDI (Tariq et al., 2016)
  o orientation dispersion index (ODI) associated with the spatial configuration of neurite structures
  o intracellular volume fraction (fICVF) associated with neurite density
– three rs-fMRI measures
  o weighted Degree Centrality (wDeCe) describing whole-brain, global connectivity
  o regional homogeneity (ReHo) describing local connectivity, i.e., synchrony of local neuronal populations (Zang et al., 2004)
  o fractional amplitude of low-frequency fluctuations (fALFF) reflecting the extent of spontaneous neural activity of the brain (Zang et al., 2007)
– T1w/T2w ratio as a measure of myelin (Glasser and Van Essen, 2011; Filip et al., 2020a, 2020b)
– volumes of structures of interest.

A cohort continuous over the whole adult age span was examined to describe age-related brain tissue alterations, with the following specific aims:

(1) Plot the age-related courses of individual qMRI parameters over main regions of interest (ROIs), enabling us to evaluate the plausibility of their use in future ageing research considering the specific neurobiological hypotheses.
(2) Perform cross-correlations between individual metrics in the main ROIs to assess eventual similarities in Aim 1 outputs quantitatively.
(3) Evaluate the unique contributions of individual protocols in main large ROIs (subcortical grey matter [GM], cerebellar GM, cortical GM and whole white matter [WM]), i.e., the ability of the MRI metric to detect a process not visible with the other MRI metrics.

Our main hypothesis presumed that individual qMRI parameters will exhibit different age curves, with different plateaus and increasing/decreasing trendlines, Furthermore, the ability of rotating frame relaxations to detect further information from much lower frequencies in the kHz range of the effective fields will give them edge over other metrics.

## Methods

### Subjects

116 volunteers were screened for this study. Basic demographic information was collected, including the Brief Questionnaire Regarding Severity of Memory & Emotional Problems (BQRS-M&E; Reisberg, 2013)—a simple, screening, patient-facing 5-item scale providing subjective assessment of eventual cognitive problems. The following exclusion criteria were utilised: MRI contraindications, claustrophobia, significant space occupying or vascular lesion in the MRI scan, psychiatric or neurologic disorder, subjectively perceived cognitive problems (BQRS-M&E ≥ 3). Every participant provided a written informed consent in accordance with the Declaration of Helsinki. The study protocol was approved by the ethics committee of the University Hospital of St. Anne, Czech Republic.

### Imaging protocol and data analysis.

MRI acquisition was performed in a 3-Tesla Siemens MAGNETOM Prisma scanner using a 64-channel head–neck coil at the Central European Institute of Technology in Brno, Czech Republic. Magnetization-prepared rapid gradient echo (MPRAGE) sequence in sagittal orientation with 1.0 mm isotropic resolution, echo time (TE) 2.47 ms, repetition time (TR) 2,150 ms, inversion time (TI) of 1,100 ms, flip angle 8°, generalized autocalibrating partial parallel acquisition (GRAPPA) acceleration factor of 2 was utilised to generate a T1-weighted (T1w) image. T2-weighted (T2w) image was acquired using the SPACE sequence in sagittal orientation, with 1.0 mm isotropic resolution, TE 72.6 ms, TR 2,820 ms, GRAPPA 2. Manufacturer-implemented pre-scan normalisation algorithm was employed for both T1w and T2w scans. Diffusion-weighted imaging (DWI) was based on the following parameters: 1.8 mm isotropic resolution, TR 2,820 ms, TE 726 ms, MB acceleration factor 4, 93 directions with 7 additional non-diffusion weighted (b0) images, two b-shells of 750 and 1,500 s/mm^2^. Two DWI acquisitions were performed with opposite phase encoding polarity [anterior–posterior (AP) followed by posterior–anterior (PA)]. T2*-weighted scans sensitive to the blood oxygenation level dependent contrast were used for the rs-fMRI acquisition, with gradient-recalled echo (GRE) echo-planar imaging (EPI) sequence, 3.0 mm isotropic resolution, TR 900 ms, TE 30.0 ms, flip angle 45°, MB acceleration factor 4, interleaved acquisition, consisting of 502 volumes. Rotating frame relaxation measurements (adiabatic T1ρ, T2ρ and RAFF4) were obtained with voxel size of 1.6 × 1.6 × 3.6 mm^3^, GRAPPA 3, TE 3.18 ms and TR = 2,000 ms, 36 slices in total. Hyperbolic secant pulses were utilised for adiabatic T1ρ and T2ρ with adiabaticity factor R = 10, pulse duration of 6 ms, bandwidth 1.3 kHz, peak power ω1^max^/(2π) of 800 Hz, and number of pulses 0, 4, 8, 12, and 16. In RAFF4 acquisitions, pulse duration was 4.56 ms, number of pulses 0, 4, 8, 12, 16 and ω1^max^/(2π) 327 Hz. No medication was administered for the MRI scanning session.

The image processing pipeline for structural T1w and T2w images was based on the human connectome project (HCP) minimal pre-processing pipeline (Glasser et al., 2013) with minor modifications. Specifically, the PreFreeSurfer step did not include the optional gradient non-linearity and B_0_ field inhomogeneity correction. The FreeSurfer step was based on the CUDA (Compute Unified Device Architecture)-enabled version of FreeSurfer 6.0^1^. PostFreeSurfer step was implemented without any changes.

The analysis of rs-fMRI acquisitions was also performed using the HCP minimal pre-processing pipeline—concatenated transformation consisting of gradient non-linearity correction, motion correction, correction of the B_0_ field inhomogeneities using the spin-echo fieldmaps, rigid-body co-registration to the T1w native space with spline interpolation, followed by the mapping to the Connectivity Informatics Technology Initiative (CIFTI) grey ordinates space using ribbon-constrained approach and regularisation with 2-mm full width at half maximum (FWHM) surface and subcortical volume smoothing. The subsequent processing utilised the HCP rsfMRI pipeline (Smith et al., 2013), which includes MELODIC independent component analysis (ICA), automatic artefact components identification *via* the FIX algorithm (Salimi-Khorshidi et al., 2014) and regressing out motion-related time-courses. HCP training data was used for the automatic classification algorithm with subsequent manual correction of the output and “non-aggressive” regression of the contribution of artefactual components out of the rsfMRI data. The AFNI package (Cox, 1996) was utilised to calculate voxel-wise weighted degree centrality (DeCe) with sparsity threshold of 0.1 (Craddock and Clark, 2016). Furthermore, Regional homogeneity was calculated as Kendall’s W over 28 neighbouring voxels (Zang et al., 2004) and fALFF as the total power within the frequency range between 0.01 and 0.1 Hz (Zang et al., 2007).

Diffusion weighted imaging processing also followed the HCP minimal pre-processing pipeline, including the optional gradient non-linearity correction. Afterwards, the output coregistered to the T1w native space of the subject was utilised for NODDI parameter calculation (Tariq et al., 2016), specifically orientation dispersion index and intracellular volume fraction.

The processing of T1ρ, T2ρ and RAFF4 maps utilised one pipeline: after 3D rigid-body motion correction of all the acquired scans to the first scan of each of these sequences (trilinear interpolation, mutual information as cost function followed by an optimization pass with sinc interpolation as implemented in the FSL 6.0 MCFLIRT), relaxation time constants were calculated utilising 2-parameter non-linear fitting (custom routines in MATLAB R2016a; MathWorks, Inc., Natick, MA). Afterwards, each map was co-registered to the T1w native space of the subject—mri_robust_register-initialized BB-register algorithm.

Visual inspection and evaluation of root-mean-squared voxel displacement for motion-correction in T1ρ, T2ρ and RAFF4 maps reconstruction, rs-fMRI and DWI data was performed to exclude subjects with framewise motion exceeding the extent of two voxels and/or substantial anatomical deviations.

**Figure 1:**
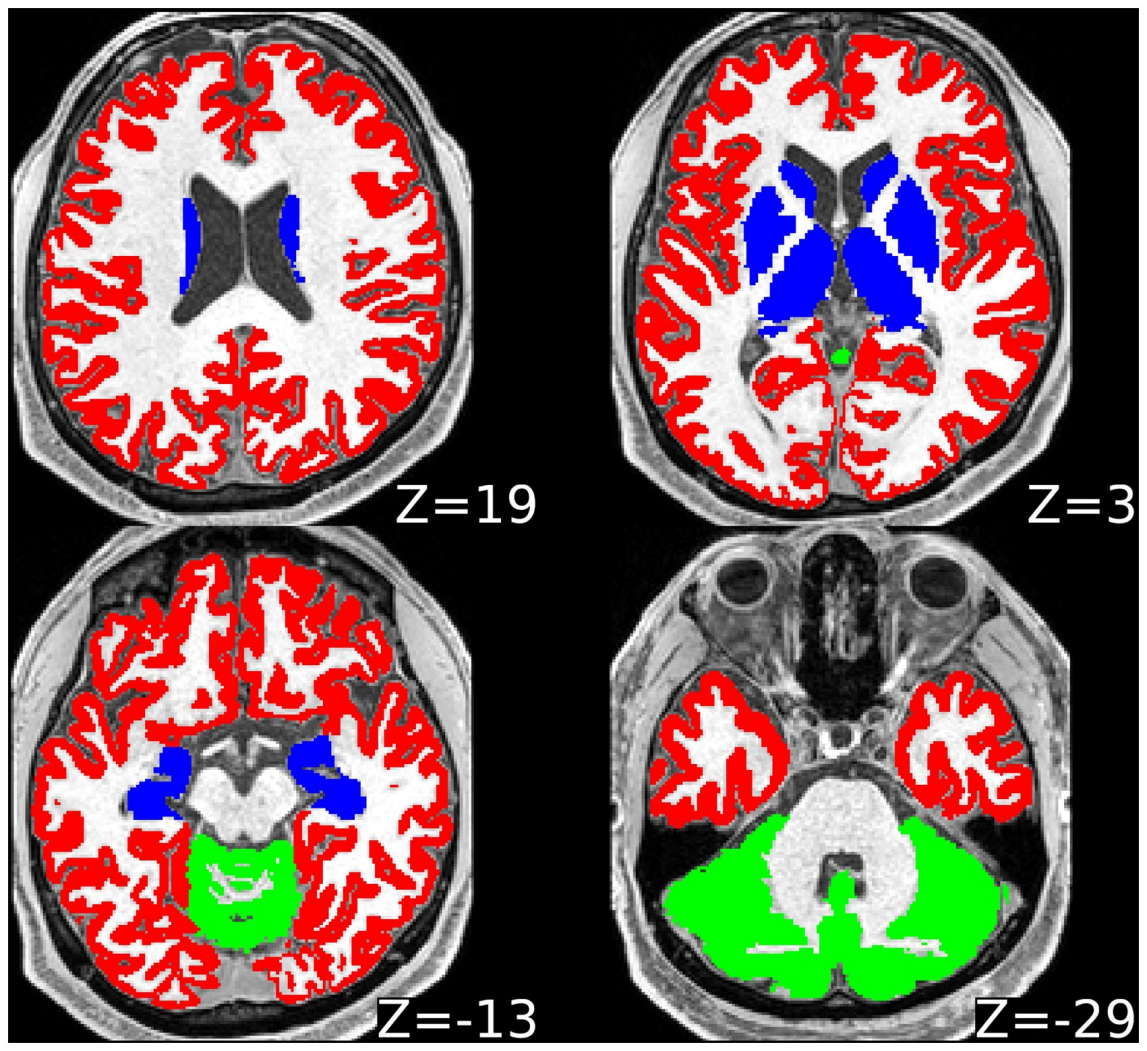
Visualisation of regions of interest from one representative subject in axial plane. Slice coordinates in the *z* axis provided, with zero reference level in the middle of the anterior commissure and posterior commissure line. Red = cortical grey matter; blue = subcortical grey matter; green = cerebellar cortical grey matter. White matter region of interest not marked in a specific colour.

And lastly, predetermined ROI masks derived from automatic FreeSurfer segmentation (cortical GM encompassing all the cerebral lobes; subcortical GM [thalamus, striatum, pallidum, hippocampus, amygdala, nucleus accumbens], cerebellar GM and whole WM) were co-registered to the images with lower resolution to avoid oversampling and inherently related problems. Brainstem was not considered in the presented analyses due to the complex structure combining GM and WM and lack of individual subsegmentation output of FreeSurfer for this area. See Figure 1 for the visualisation of GM ROIs.

All data processing outputs were visually evaluated by trained operators (VK and PF).

Medians of individual MRI metrics of interest (T1ρ, T2ρ, RAFF4, ODI, fICVF, T1w/T2w ratio, wDeCe, ReHo, fALFF) were calculated over individual ROIs in each subject. ROI volumes were normalised using the estimated intracranial volume. For the Aim 2, rank full correlation (Spearman’s correlation coefficient) between individual MRI metrics was calculated, and for Aim 3, both rank full and partial correlation of age and individual MRI metrics was calculated, controlling each time for all the other MRI metrics in the latter case and the effect of sex. False discovery rate (FDR) correction was implemented: in Aim 2 over individual ROIs, and in Aim 3 over full and partial correlation analysis separately (Benjamini and Hochberg, 1995). Alpha of 0.05 was utilised as the type I error threshold.

## Results

Seven subjects were excluded in total due to incomplete acquisition and/or excessive motion, leaving 109 subjects for further analyses.

Demographic and BQRS-M&E data was lost in one subject. Furthermore, based on the evaluation of BQRS-M&E data, four more subjects were excluded, leaving 104 subjects (51 female; median age 44.5 years, age range 19–89) in total for the final analysis. For further demographic information, including body weight, height, body mass index, education level, alcohol, tobacco and medication use, see the Supplementary Table S1. For the overview of age distribution, see the Supplementary Figure S1.

**Figure 2:**
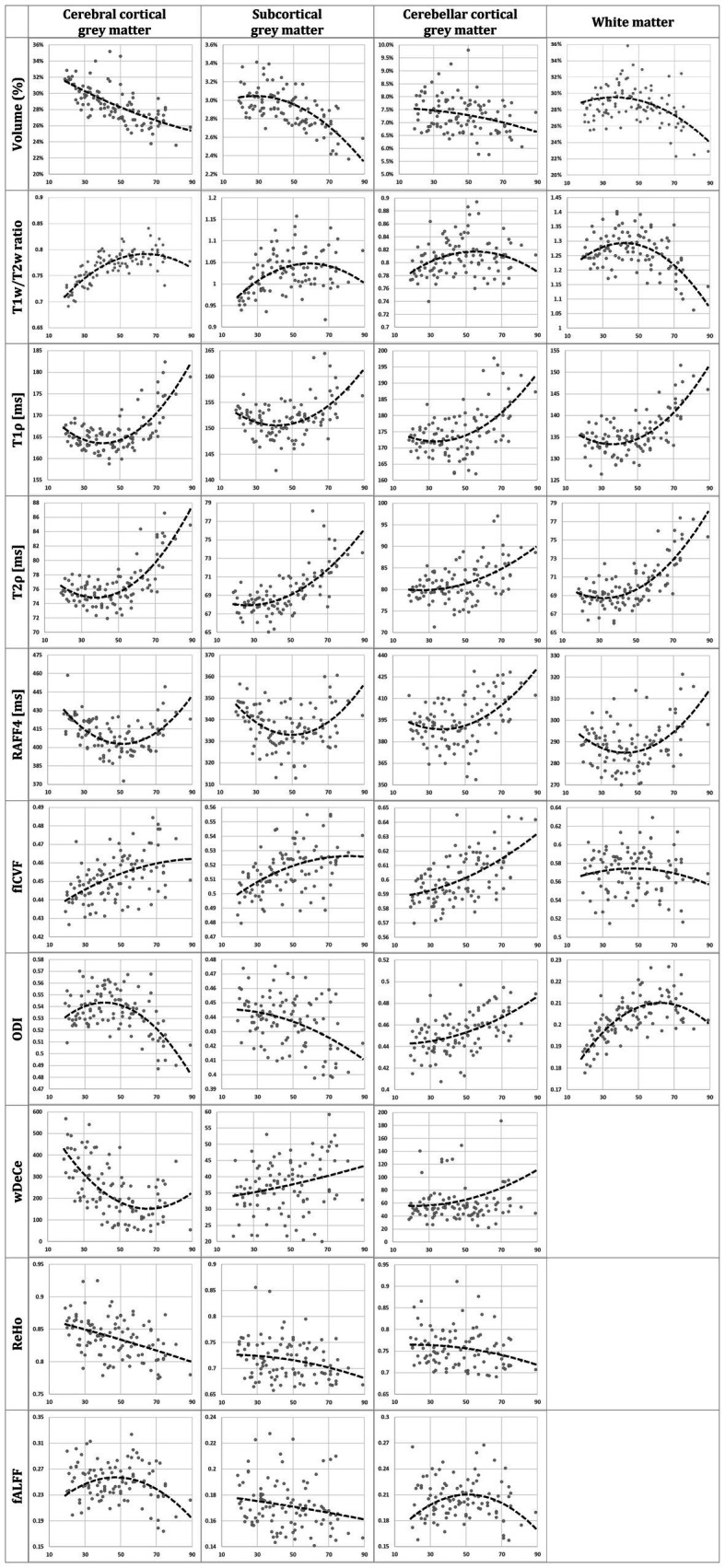
Scatterplots of age in years (axis *x*) and individual MRI metrics (axis *y*): normalised volume, T1w/T2w ratio, T1ρ, T2ρ, rank 4 Relaxation Along a Fictitious Field (RAFF4), intracellular volume fraction (fICVF), orientation dispersion index (ODI), weighted Degree Centrality (wDeCe), regional homogeneity (ReHo), and fractional amplitude of low-frequency fluctuations (fALFF), for each of the four regions of interest (cerebral cortical grey matter (GM), subcortical GM, cerebellar cortical GM and white matter [not provided for resting-state fMRI metrics]). Second order polynomial trendline is overlaid in each scatterplot.

**Figure 3:**
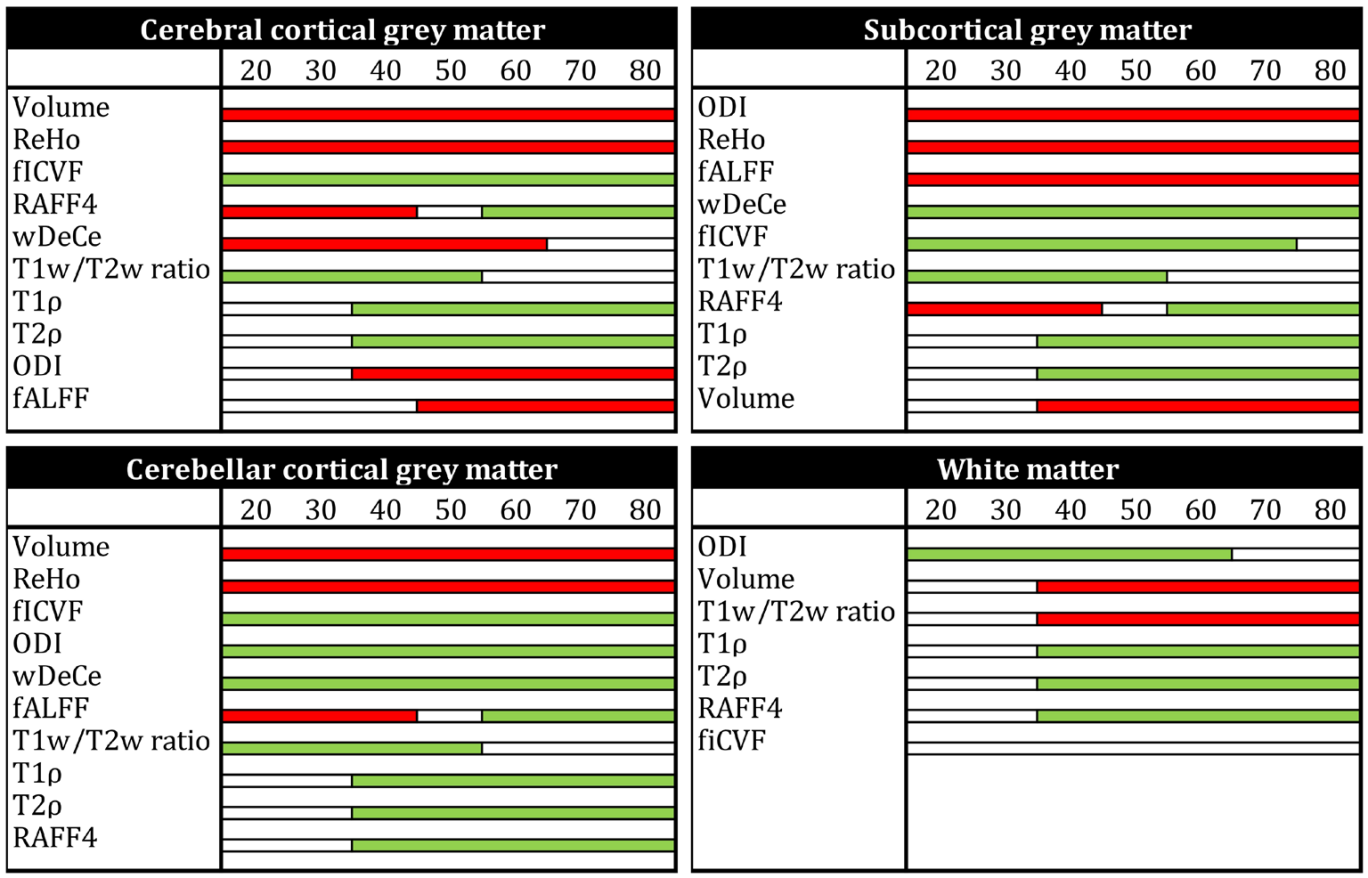
Simplified overview of ages when individual MRI metrics are able to detect underlying changes (in ascending order), for each of the four regions of interest [cerebral cortical grey matter (GM), subcortical GM, cerebellar cortical GM and white matter (not provided for resting-state fMRI metrics)]. Normalised volume, T1w/T2w ratio, T1ρ, T2ρ, rank 4 Relaxation Along a Fictitious Field (RAFF4), intracellular volume fraction (fICVF), orientation dispersion index (ODI), weighted Degree Centrality (wDeCe), regional homogeneity (ReHo), and fractional amplitude of low-frequency fluctuations (fALFF) are presented. Red colour or the bar denotes a descending trend of the MRI metric, green colour an ascending trend and white bar marks plateau/no change in the respective age group.

The results for Aim 1, scatterplots of individual MRI parameters and ages over predetermined ROIs, are presented in the Figure 2 showing individual scatterplots (axis x representing the relevant metric, axis y representing age in years) with overlaid 2nd order polynomial trendlines. Figure 3 provides a simplified overview of visually estimated ages when individual qMRI metrics detect some underlying changes, ordering the qMRI metrics based on this parameter.

**Figure 4:**
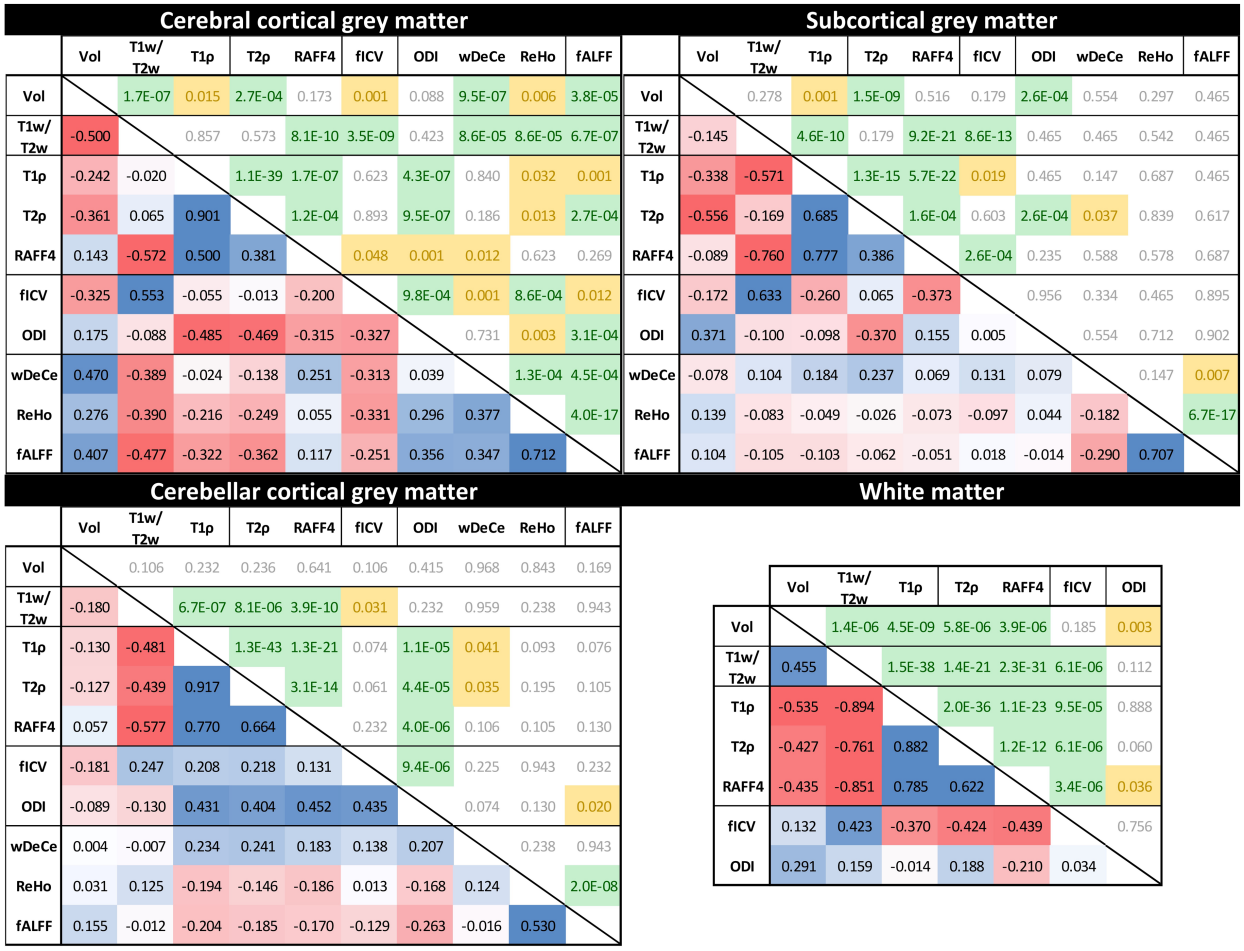
Rank cross-correlation matrices between individual MRI metrics across the four predetermined regions of interest [cerebral cortical grey matter (GM), subcortical GM, cerebellar cortical GM and white matter (not provided for resting-state fMRI metrics)]. Numbers in the lower triangle represent Spearman’s correlation coefficients (positive correlations in blue and negative correlations in red). The upper triangle of each matrix contains False discovery rate-corrected *p*-values (*p*-values < 0.001 in green, *p*-values between 0.001 and 0.05 yellow).

Figure 4 provides the output for the Aim 2—analysis of cross-corelation between individual MRI metrics—for all the predetermined ROIs. The lower triangle of presented matrices states relevant Spearman’s correlation coefficients, marking positive correlations in blue and negative correlations in red. The upper triangle of each matrix contains FDR-corrected (separately for each of the four ROIs) *p*-values, marking p-values < 0.001 in green and p-values in the range of 0.001–0.05 yellow for better visibility.

And lastly, the results for Aim 3, Spearman’s correlation analysis of age and MRI metrics, are presented in the Table 1. Full rank correlation coefficients, with respective FDR-corrected p-values, and partial rank correlation coefficients after controlling for the remaining MRI metrics of interest, with respective FDR-corrected p-values, are shown.

**Table 1:** Full and partial rank correlations between the age and individual MRI metrics in the four predetermined regions of interest [cerebral cortical grey matter (GM), subcortical GM, cerebellar cortical GM and white matter (not provided for resting-state fMRI metrics)].

	Full correlation				Partial correlation			
	Cerebral cortical GM	Subcortical GM	Cerebellar cortical GM	White matter	Cerebral cortical GM	Subcortical GM	Cerebellar cortical GM	White matter
Volume	−0.709* **(4.31E-17)**	−0.579* **(1.44E-10)**	−0.284* (0.004)	−0.184 (0.081)	−0.307* (0.009)	−0.178 (0.171)	−0.165 (0.166)	−0.157 (0.123)
T1w/T2w ratio	0.708* **(4.31E-17)**	0.385* **(5.70E-05)**	0.177 (0.070)	−0.228* (0.032)	0.454* **(1.83E-05)**	0.205 (0.131)	0.437* **(5.03E-05)**	0.328* (0.001)
T1ρ	0.393* **(3.97E-05)**	0.247* (0.012)	0.480* **(2.67E-07)**	0.477* **(6.26E-07)**	0.124 (0.309)	0.121 (0.283)	0.049 (0.628)	0.301* (0.003)
T2ρ	0.486* **(1.93E-07)**	0.625* **(1.69E-12)**	0.460* **(8.77E-07)**	0.603* **(4.17E-11)**	0.155 (0.243)	0.430* **(7.10E-05)**	0.102 (0.386)	0.268* (0.008)
RAFF4	−0.205* (0.037)	−0.093 (0.342)	0.422* **(8.51E-06)**	0.166 (0.107)	−0.026 (0.797)	−0.101 (0.352)	0.300* (0.008)	0.044 (0.656)
fICVF	0.484* **(2.17E-07)**	0.486* **(1.93E-07)**	0.549* **(1.77E-09)**	−0.039 (0.715)	0.168 (0.234)	0.344* (0.002)	0.306* (0.008)	0.204* (0.047)
ODI	−0.347* **(3.20E-04)**	−0.365* **(1.43E-04)**	0.550* **(1.77E-09)**	0.648* **(4.42E-13)**	−0.080 (0.533)	−0.149 (0.195)	0.208 (0.093)	0.615* **(9.05E-12)**
wDeCe	−0.525* **(1.21E-08)**	0.343* **(3.61E-04)**	0.222* (0.024)	**–**	−0.293* (0.010)	0.166 (0.171)	0.068 (0.552)	**–**
ReHo	−0.450* **(1.65E-06)**	−0.215* (0.028)	−0.224* (0.023)	**–**	0.066 (0.569)	−0.032 (0.750)	−0.105 (0.386)	**–**
fALFF	−0.585* **(8.47E-11)**	−0.247* (0.012)	−0.382* **(6.50E-05)**	**–**	−0.144 (0.253)	−0.163 (0.171)	−0.187 (0.123)	**–**

Full correlations were controlled for sex only; partial correlations were controlled for all the other remaining MRI metrics of interest and sex. False discovery rate-controlled *p*-values are provided in parentheses after Spearman’s correlation coefficients. Asterisks mark statistically significant results; bold highlighted results provide *p*-values < 0.001. Grey values are not statistically significant.

## Discussion

This is the first study providing a complex overview of research-focused qMRI modalities, which are still relatively simple to implement and potentially useful in ageing research. Unsurprisingly, subject age is associated with a substantial effect on MRI metrics and all MRI protocols utilised in this study provided statistically significant results in one or more ROIs. However, the interpretation of their findings is far from straightforward, pointing to the presence of multiple elusive, latent variables.

Previously published histological validations or presumed models of tissue structure which individual MRI metrics (Michaeli et al., 2009; Mitsumori et al., 2009; Hakkarainen et al., 2016; Tariq et al., 2016) are supposed to be sensitive to do not fully explain the shapes of trendlines overlaid over the scatterplots in Figure 2. The use of 2nd order polynomial trendlines is definitely an oversimplification, but a logical one in the least. They fit rather well the presumed plateau corresponding to the relatively stable health and performance in early adulthood, followed by sharper decline in higher age, sometimes hypothesised to reach even exponential rate in some areas (Cox, 1996; Salimi-Khorshidi et al., 2014). For volume trendlines, this hypothesis seems to hold well for subcortical GM and WM, with sharper decline of volumes after the age of 50. However, cerebral and cerebellar cortical GM volume seem to decline more linearly over the whole age span acquired in the study.

While the interpretation of volume alteration is rather straightforward, the situation is more complex in further metrics. T1w/T2w ratio, previously labelled “myelin map” for cerebral cortical GM, since areas with higher T1w/T2w values corresponded to regions with higher myelin content (Glasser and Van Essen, 2011), fails to follow the age-related development of the underlying biology as seen in electron microscopy. While myelination is thought to continue well into early adulthood, normal ageing has been repeatedly associated with the loss of myelin integrity such as formation of splits and myelin balloons directly correlated with cognitive decline (Craddock and Clark, 2016). To add to the complexity of the process, oligodendroglial cells in the cerebral cortex seem to be unable to sustain myelin sheath quality, leading to decrease of internodal lengths of myelin sheaths, structural alterations with the presence of degenerating oligodendroglial cytoplasm in some sheaths and concomitant production of dysfunctional redundant myelin lamellae (Peters, 2002; Peters and Sethares, 2003). Since these very myelin lamellae are thought to be a substantial contributor to MRI signal attributed to myelin, we are hesitant to explain the rise in T1w/T2w ratio in the cerebral cortical GM as clear proof of the increase of myelination in its true meaning. Strangely though, although T1w/T2w ratio as “myelin map” was validated only in cerebral cortex, the shape and position of its trendline in the white matter in the Figure 2 may be considered to correspond much better to the presumed biology, despite well substantiated doubts about the association of this metric with white matter myelin concentration (Uddin et al., 2018). And lastly, while our T1w/T2w ratio age scatterplots are not in complete discord with previous studies (Grydeland et al., 2019), they fail to follow the shapes of other metrics previously validated as myelin markers both in histological (Satzer et al., 2015) and clinical studies in patients with demyelinating disorders (Filip et al., 2020a, 2020b, 2021)—RAFF4 and T1ρ. RAFF4 exhibits generally U-shaped curves, with low inter-individual variability in individual age groups mainly in cerebral cortical GM. However, as seen in the Table 1, this rather conspicuous and believable development of a plateau around the age of 50 is associated with only modest correlation and statistical significance.

The very same issue seems to haunt also MRI metrics hypothesised to be associated with cellular membrane integrity or cellularity in general—fICVF, T1ρ and partly also ODI. In all four ROIs, T1ρ seems to correspond rather well to the overlaid second order polynomial trendline, with steep increase after the age of 50–60, preceded by a mild decrease or a plateau stage. fICVF, i.e., fraction of the intracellular volume over the sum of intra-and extracellular volumes, rises rather steadily in all GM types throughout the age span, without sharp direction changes. Even its cross-correlation with T1ρ is borderline at best. On the other hand, ODI corresponds rather well to the ageing milestones in the cerebral cortical GM detected by T1ρ. However, cerebellar cortical GM exhibits a dissimilar shape in ODI than cerebral cortical GM, pointing to the selectivity of this method to non-negligible differences in cerebral and cerebellar ageing (Filip et al., 2019; Filip and Bareš, 2021). Moreover, ODI, as the only metric of the studied parameters, detects an age-related process leading to steep change in WM characteristics in youth followed by a plateau or mild decrease after the age of 50. This unique sensitivity is also confirmed by partial correlation analysis in WM in the Table 1 and in the Figure 3, where ODI shows no or only minimal cross-correlations with other MRI metrics in WM, making it an important candidate to consider in any ageing-focused research. Nonetheless, the most striking discrepancy in these findings is the failure to replicate the microscopic findings: a mild loss of cortical neurons has been suggested in normal ageing, but nowhere as extensive as suggested in early reports (Peters et al., 1998) or to be inferred from the presented data.

Adiabatic T2ρ was the only MRI metric in this study previously suggested as an iron marker (Michaeli et al., 2007; Mitsumori et al., 2009). Iron content in the cerebral cortical GM is an especially important factor since iron load is a risk factor of several neurodegenerative diseases (Bartzokis et al., 2004). But although about 80% of neocortical iron is stored in ferritin, myelinated fibers also have an increased iron concentration in several cortical areas (Stüber et al., 2014). While this may be one of the reasons for high cross-correlations (see Figure 3) of this metric with other MRI parameters presumed to be sensitive to other biological underpinnings, the extent of similarity casts doubts on the utility of the combined use of these highly intercorrelated MRI metrics in the ageing research. Nonetheless, the very biological processes detected by these MRI protocols may well be interlinked in normal ageing, even though they are distinct in their nature and physiological processes. Ergo, by no means does the Figure 3 prove the combined use of presented MRI metrics to be futile, since other physiological or pathological circumstances may lead to uncoupling of underlying biology and therefore different sensitivity of utilised MRI protocols (Filip et al., 2020a, 2020b). Furthermore, as seen in partial correlations in the Table 1, T2ρ is definitely able to detect a unique process in subcortical GM not visible by any other MRI measures implemented in this study.

And lastly, rsfMRI metrics, by their very nature, are generally difficult to interpret and relate specific biological processes to rsfMRI alterations, since the origin of BOLD (blood oxygen level dependent) signal and its association with the underlying neural activity is still a matter of dispute (Drew, 2019). Nonetheless, a widely accepted model of BOLD and local field potential coupling assumes an indirect entrainment of BOLD by neural activity, mostly post-synaptic potentials, but not the spiking rate (Ekstrom, 2010). Although highly cross-corelated in the cerebral cortical GM, they still provide an interesting addition to the whole picture of brain ageing. The age-related decrease of global connectivity (wDeCe) in cerebral cortical GM is then strangely countered by its increase in subcortical and cerebellar cortical GM. Local connectivity (ReHo) seems to decline generally with age in all the ROIs.

Several limitations to this study need to be acknowledged. Firstly, this study lacks complex formal neuropsychological examination to exclude subjects with non-diagnosed cognitive problems. While the evaluation of subjective cognitive impairment utilised here provides some help in this matter, further studies focused mainly on subjects of higher age should be designed with this possibility in mind. And secondly, this paper generally avoids biological interpretations of the presented findings and cautions against simplistic likening of individual qMRI parameters to one underlying pathophysiology under all circumstances. Hence, even the claims on qMRI parameters utilised in this study, as stated in the introduction, must be taken with a grain of salt. While T1ρ as a compound metric has been previously associated with neuronal density, there are no studies directly distinguishing between the effect of other cells in the central nervous system on this parameter. By the same token, other metals may affect T2ρ as an additional relaxation channel from previously published iron. And lastly, NODDI parameters distinguish in general between intracellular and extracellular space, without any putative selectivity for neurons or glia. While exceedingly difficult, neuroscientific research would greatly benefit from MRI protocols able to disentangle this issue and differentiate between neurons and glia.

## Conclusion

All in all, this unique overview provides a quantitative baseline for the design of further studies and choice of relevant MRI parameters depending on the age group of interest. It clearly shows that MRI metrics are generally highly sensitive, but very poorly specific tools. One must even consider the possibility that the main biological underpinnings of some MRI metrics may be age-dependent as well -one biological process may be the primary driver of the detected signal in early age, but later end up overshadowed by another process developing only in higher age–a process which could explain the U-shape of curves in RAFF4. Even partial correlations do not show whether the MRI metrics detect the same biology or interlinked, but distinct microstructural features, since ageing-related cascades often consist of concomitant and co-dependent biological processes of substantially different nature and effect on MRI signal. Therefore, the newly emerging field of *in vivo* histological MRI aiming to uncover the true underlying biology inherently requires multimodal approach with advanced post-processing models combining several metrics, which would extract the dominant, shared subcomponent with much higher microstructural specificity than each metric individually. Furthermore, very careful multimodal validation studies against biological tissue samples, ranging from simplified phantoms to true biological specimen in combination with extensive histological and microscopic evaluation, must be considered to achieve the full potential of novel MRI approaches.

## Data availability statement

Raw or processed data of this study are not publicly available due to the sensitive nature of human data acquired in patients. The data is available upon reasonable request to the corresponding author.

## Ethics statement

The studies involving human participants were reviewed and approved by Ethics Committee of the University Hospital of St. Anne, Brno, Czechia. The patients/participants provided their written informed consent to participate in this study.

## Author contributions

PF participated in the design of the study, performed the data analysis and wrote the manuscript. VK recruited the study subjects, performed the blinded quality control and reviewed the manuscript. ZV was consulted about the statistical methods and reviewed the manuscript. MB recruited the study subjects and reviewed the manuscript. SiM and ShM participated in the design of the study and reviewed the manuscript. LV designed the acquisition protocol, monitored data acquisition, and reviewed the manuscript. All authors contributed to the article and approved the submitted version.

## Funding

Financial support for this project was provided by the General University Hospital in Prague (MH CZ-DRO-VFN64165). We acknowledge the core facility MAFIL of CEITEC supported by the MEYS CR (LM2018129 Czech-BioImaging). We also acknowledge the National Institutes of Health for support to SiM and ShM (Funding P41 EB027061 and R01 AG055591).

## Conflict of interest

The authors declare that the research was conducted in the absence of any commercial or financial relationships that could be construed as a potential conflict of interest.

## Publisher’s note

All claims expressed in this article are solely those of the authors and do not necessarily represent those of their affiliated organizations, or those of the funding agencies, the publisher, the editors and the reviewers. Any product that may be evaluated in this article, or claim that may be made by its manufacturer, is not guaranteed or endorsed by the publisher.

## Supplementary material

The Supplementary material for this article can be found online at: https://www.frontiersin.org/articles/10.3389/fnagi.2023.1099499/full#supplementary-material

## Footnotes

^1^ http://surfer.nmr.mgh.harvard.edu

## Abbreviations

MRI: magnetic resonance imaging
rsfMRI: resting-state functional MRI
qMRI: quantitative MRI
T1w: T1-weighted
T2w: T2-weighted
DWI: diffusion-weighted imaging
RAFF4: Relaxation Along a Fictitious Field of rank 4
ODI: orientation dispersion index
fICVF: intracellular volume fraction
wDeCe: weighted Degree Centrality
ReHo: regional homogeneity
fALFF: fractional amplitude of low-frequency fluctuations
ROI: region of interest
GM: grey matter
WM: white matter
