# Supplementary figures and images for: Utility of quantitative MRI metrics in brain ageing research
Source: Front Aging Neurosci. 2023 Mar 9;15:1099499. doi: 10.3389/fnagi.2023.1099499 (PMC10034010; doi:10.3389/fnagi.2023.1099499)

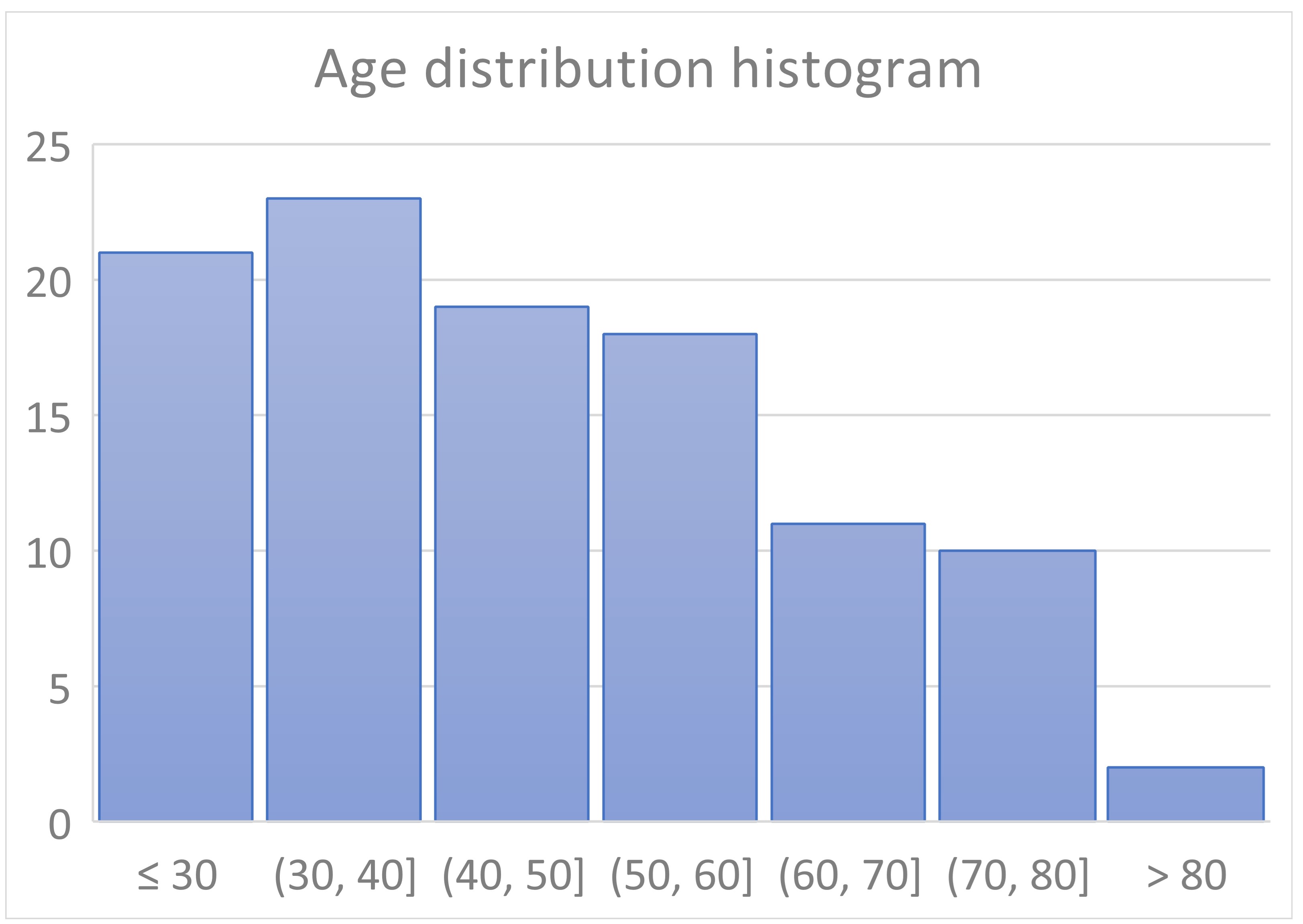

Supplement: Supplementary file 2 [file Image_1.JPEG]
